# Extraction of long-term tine-based leadless pacemakers through the jugular approach: two case reports

**DOI:** 10.1093/ehjcr/ytaf600

**Published:** 2025-11-26

**Authors:** Alexander Breitenstein, Nadine Molitor, Daniel Hofer

**Affiliations:** Department of Cardiology, University Heart Center, University Hospital Zurich, Raemistrasse 100, Zurich 8091, Switzerland; Department of Cardiology, University Heart Center, University Hospital Zurich, Raemistrasse 100, Zurich 8091, Switzerland; Department of Cardiology, University Heart Center, University Hospital Zurich, Raemistrasse 100, Zurich 8091, Switzerland

**Keywords:** Case report, Leadless pacemaker, Retrieval, Jugular route

## Abstract

**Background:**

Tine-based leadless pacemakers (LP) have been in clinical use for over a decade. However, optimal management at the time of battery depletion remains uncertain. In this report, we describe the successful extraction of two tine-based LP, performed 4 and 8 years after initial implantation, using a right internal jugular venous approach.

**Case summary:**

Two patients (86-year-old man and a 93-year-old woman) received their initial tine-based LP four and 8 years ago, respectively, for the prevention of severe bradycardia in the setting of permanent atrial fibrillation. Both underwent implantation of a new device due to battery depletion via a right jugular approach. After successful deployment, a steerable sheath and a Goose Neck Snare were used to retrieve the old device. This was successful in both cases and the previously implanted device could be extracted with little resistance.

**Conclusion:**

Retrieval of tine-based LP is possible and safe even after long-term implantation.

Learning points

- Extraction of tine-based leadless pacemakers is feasible, even after long-term dwell times.
- The jugular approach provides an alternative route for retrieval, using the same tools typically employed via the femoral access.

## Introduction

The tine-based leadless Micra^TM^ Transcatheter Pacing System (TPS; Medtronic, Minneapolis, MN) have been in clinical use for over a decade. However, optimal management at the time of battery depletion remains uncertain. In many cases, the standard approach is to leave the existing device in place and implant a new leadless pacemaker (LP) adjacent to it. Nevertheless, certain clinical scenarios—such as suspected device infection or young patient age—may warrant evaluation of extractability even with longer dwell times. The jugular approach has been established as an alternative approach for LP implantation, but its merit for extraction has not been evaluated. In this report, we describe the successful extraction of two tine-based LP, performed 4 and 8 years after initial implantation, using a right internal jugular venous approach.

## Summary figure

**Figure:**
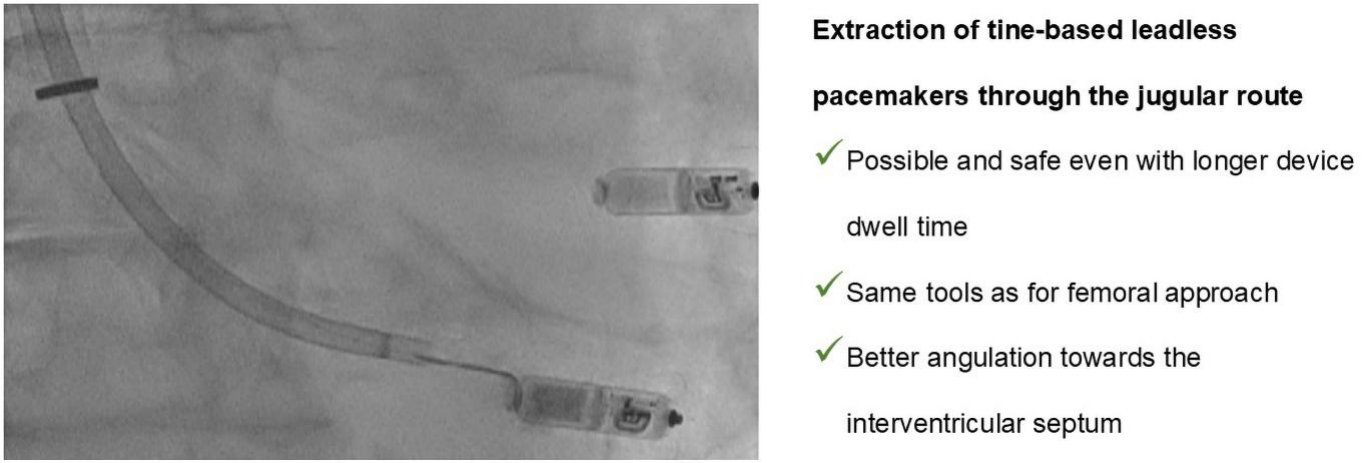


## Case presentation

### Patient 1

This 86-year-old man received his initial tine-based LP 4 years ago for the prevention of severe bradycardia in the setting of permanent atrial fibrillation following multiple left atrial catheter ablations. Echocardiography revealed preserved left ventricular function (LVEF 57%) but significantly enlarged right (72 mL/m²) and left atrial volume index (55 mL/m²). His medical history was negative for prior cardiac surgeries; however, he had comorbidities including chronic obstructive pulmonary disease, arterial hypertension, and chronic kidney disease. Due to a high pacing burden and suboptimal ventricular capture threshold, device replacement became necessary earlier than anticipated.

**Figure 1:**
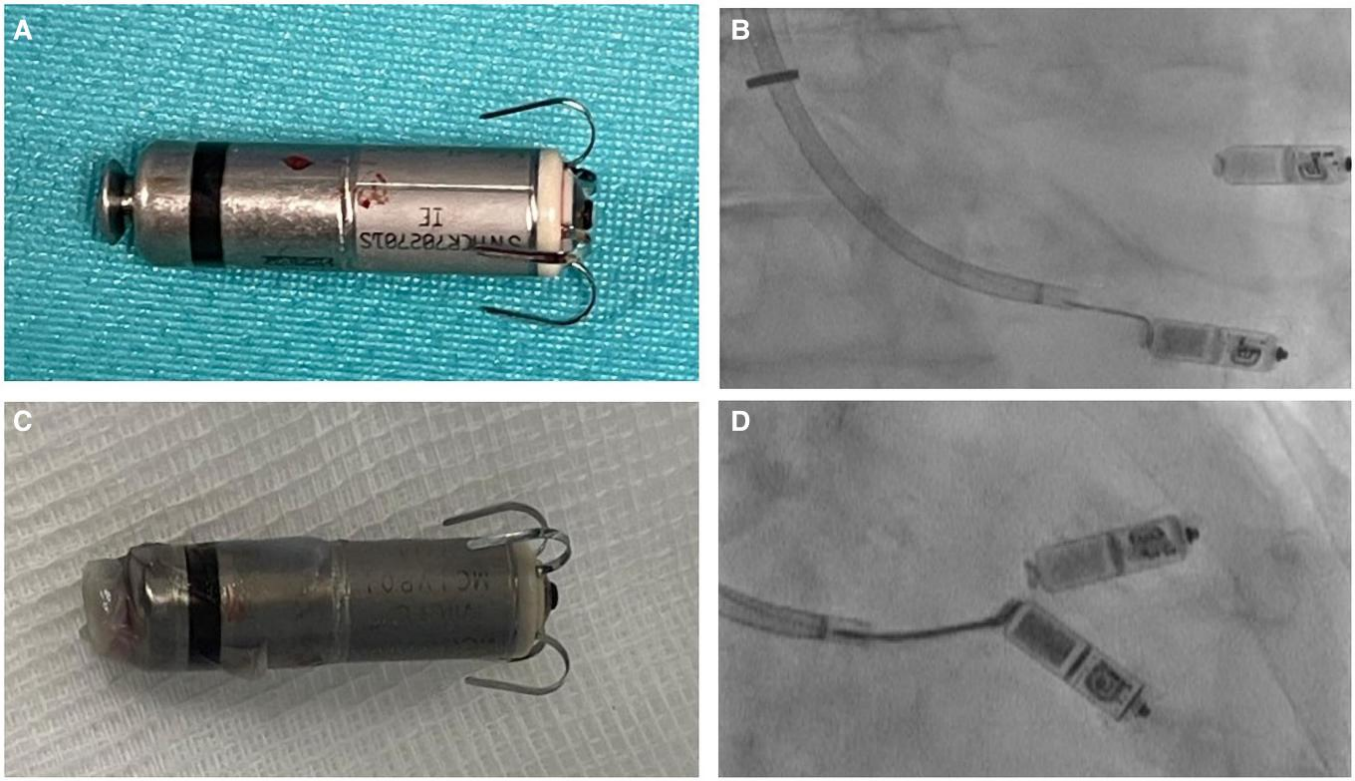
(*A*) The Goose Neck Snare encircled the retrieval button of the device. (*B*) No adhesive tissue surrounding the extracted device. (*C*) Retrieval button engaged by the Goose Neck Snare. (*D*) Some amount of encapsulating tissue at the proximal part of the device.

Given the markedly dilated atria, a jugular venous approach was deemed favourable for implantation. After discussion with the patient, we also planned to attempt extraction of the old device during the procedure. Following standard disinfection and sterile draping of the surgical site, the new LP was implanted in the usual manner. After removal of the delivery tool, an 8.5 Fr steerable large-curl sheath (Agilis, Abbott, Chicago, IL) was introduced into the right atrium over the 27 Fr introducer sheath (Medtronic) from the new implantation. A 10 mm Goose Neck Snare (Medtronic) was then advanced through the sheath to its distal end. Both the sheath and snare were navigated across the tricuspid valve toward the retrieval button of the previously implanted device. The snare easily engaged the body of the pacemaker (*Video 1A*) and was carefully withdrawn until it encircled the retrieval button (*Figure 1A*, *Video 1B*). Once secured, the snare was closed, and the device was gently extracted from the myocardium using steady traction (*Video 1C*). Following successful removal, the sheath was withdrawn from the patient's body, and the venous access site was closed using a modified figure-of-eight suture. Device inspection confirmed no adhesive tissue surrounding the extracted device (*Figure 1B*).

### Patient 2

The second patient was a 93-year-old woman with ischaemic cardiomyopathy and a history of multiple percutaneous coronary interventions. Due to challenges with medical rate control for her permanent atrial fibrillation, she underwent atrioventricular (AV) node ablation and implantation of a tine-based LP (Micra^TM^ VR, Medtronic) in 2017. Given her elevated thromboembolic risk (CHA₂DS₂-VASc score of 6), she has been maintained on oral anticoagulation with rivaroxaban. She was referred for evaluation due to early battery depletion, attributing to a gradual increase in her ventricular pacing threshold over recent years. Similar to the first case, a new tine-based pacemaker was implanted via the right internal jugular vein. At the end of the procedure, an 8.5 Fr steerable sheath (Agilis large-curl, Abbott) was introduced together with a 25 mm Goose Neck Snare (Medtronic) to capture the old device. The snare engaged the body of the pacemaker nearly to its distal end (*Video 2A*), suggesting minimal fibrous adhesion. It was then carefully pulled back to the retrieval button (*Figure 1C*, *Video 2B*), closed securely around it, and the device was gently detached from the ventricular myocardium with controlled traction (*Video 2C*). The pacemaker was then withdrawn into the right atrium and explanted via the large-bored introducer sheath. Interestingly, the proximal body of the Micra^TM^ device showed some encapsulating tissue during inspection after extraction (*Figure 1D*).

## Discussion

LP technology provides an effective treatment option for bradyarrhythmias while eliminating the long-term complications associated with transvenous leads.^1,2^ Despite the promising performance of LP, there are still no standardized recommendations for end-of-service (EOS) management.^3^ The design and programming capabilities of the tine-based Micra™ (Medtronic, Minneapolis, USA) device allow it to be switched into a nonfunctional mode at EOS, enabling the device to be safely left in place. Given the advanced average age of most patients receiving a Micra™ implant,^2^ this approach is often both practical and of low risk. However, certain clinical scenarios may favour device retrieval over abandonment, such as suspected device infection, younger patient age, patient wish, or the need to minimize device burden in the right ventricle.^2–8^ In such scenarios, attempting extraction of a Micra™ device is a viable option and should be discussed with the patient, carefully weighing the potential benefits and risks. Although some reports have described encapsulation of the device body even after relatively short dwell times,^9,10^ available data remain limited, and the extent of encapsulation appears highly variable. However, the published data so far have demonstrated a high success rate of Micra^TM^ extraction with a dwell time of up to 9 years with a low major complication burden.^7,11–15^ Retrieval can be performed using a steerable sheath and a single-/multiple loop snare^11,12^ but also dedicated retrieval tools are available these days (AVEIR Retrieval Catheter System, Abbott).^15^ Moreover, the advantages of leadless device implantation via the jugular route such as easier traversal of the tricuspid valve and a more perpendicular approach to the interventricular septum also apply to the retrieval procedure.^16^ During the two procedures, several technical insights became evident for future retrievals:

1. Tool selection and procedural technique do not require modification when compared to the standard femoral approach.
  - As in the cases presented herein, a steerable sheath can be used together with a single- or triple-loop snare.
  - The introducer catheter from the concomitantly device implantation could be used together with a small single- or triple-loop snare.
  - A dedicated retrieval catheter (Abbott AVEIR^TM^ steerable retrievable catheter) has been introduced, which can be used as well to retrieve a previously implanted LP independently from the manufacturer.
2. Snaring difficulties occurred in the second case, where the snare loop engaged the newly implanted device multiple times due to the close proximity of the two LP. To avoid this, we recommend implanting the new device at a greater distance from the old one, if both technically and electrically feasible particularly when an extraction attempt is anticipated, as in the first patient.
3. Snare size matters: A 10 mm single-loop snare proved superior in handling compared to the larger 25 mm version.
4. Access route considerations: The jugular approach is likely less familiar to most operators but can offer in some cases potentially a better angle and an improved alignment with the targeted device.

Successful extraction of a tine-based LP via the right internal jugular route is safe and feasible even several years after implantation. The benefits of this attempt, however, have to be balanced vs. the risk in each individual case. Importantly, according to the published case reports and previous reports,^17^ there were no signs that the leaflets of the tricuspid valve were damaged as a result of the retrievable procedure.

## Data Availability

The data underlying this article cannot be shared publicly due to the privacy of the individuals that participated in the study. The data will be shared on reasonable request to the corresponding author.

## Lead author biography

Senior consultant in electrophysiology trained in all aspects of interventional rhythmology. Works at the University Heart Center in Zurich after a training fellowship at Barts Heart Center in London/UK.

**Consent:** All authors have contributed equally to the manuscript.

**Patient informed consent:** Both patients have given written informed consent (compliant with COPE guidelines).

**Funding:** This work was not supported by financial funding.
